# Medial Patellofemoral Ligament Reconstruction Using the Hamstring Tendon for Patellofemoral Joint Instability in an 81-Year-Old Female

**DOI:** 10.2174/1874325001711011028

**Published:** 2017-08-29

**Authors:** Tsuneari Takahashi, Katsushi Takeshita

**Affiliations:** Department of Orthopedic Surgery, Jichi Medical University, Shimotsuke, Japan

**Keywords:** Medial patellofemoral ligament, Patellofemoral instability, Hamstring tendon graft, MPFL reconstruction, Physiotherapy, Hip OA

## Abstract

**Introduction:**

Chronic patellofemoral instability occurs mainly in adolescent females and can also be induced by medial patellofemoral ligament (MPFL) injury. There are no case reports of MPFL reconstruction for chronic patellofemoral instability due to MPFL injury in aged populations.

**Case Presentation:**

81-year-old female presented with left knee pain, giving way, and patellar instability while climbing stairs, which continued for 18 months. Patellar apprehension test was positive, and roentgenogram showed lateral patellar subluxation. Conservative therapy was not successful; hence, we performed a lateral release and MPFL reconstruction surgery.

**Operative Procedure:**

After arthroscopic lateral release, the hamstring tendon was harvested, and a graft composite made of doubled hamstring tendon and polyester tape with a suspensory fixation device was prepared. Then, a femoral bone tunnel was constructed in a socket shape at the anatomical footprint of the MPFL. The graft was passed through the femoral tunnel, and free ends of the graft composite were sutured to the periosteum of the patella, using two suture anchors at 60° of knee flexion with patellar reduction. Physiotherapy was gradually started using a patella-stabilizing orthosis on the first postoperative day. Her Kujala score improved from 66 to 97 points, and Barthel index score improved from 70 to 100 points at 1 year after surgery. She neither developed patellofemoral joint OA nor had any recurrence of symptoms at the 5-year postoperative follow up.

**Conclusion:**

MPFL reconstruction using the hamstring tendon is an effective procedure for patients with chronic patellofemoral instability even after the age of 80 years.

## 1 INTRODUCTION

Chronic patellofemoral instability is a complex problem caused by bone malformation, soft tissue abnormalities, and joint laxity and occurs mainly in adolescent females. It is also induced by medial patellofemoral ligament (MPFL) injury [1-3]. The MPFL is the primary stabilizer of the patellofemoral joint (PFJ); it stabilizes the patella medially during knee motion and controls patellar tracking, providing approximately 53%–60% of restraining force [4, 5]. There are no case reports of MPFL reconstruction in elderly females. We report a rare case of PFJ instability without PFJ osteoarthritis (OA) in an elderly female who was injured by a fall from the stairs; she then underwent MPFL reconstruction and exhibited satisfactory progress.

## 2 CASE PRESENTATION

An 81-year-old female who walked with the aid of a stick presented with left knee pain, giving way of the knee, and patellar instability while climbing stairs, all of which had persisted for 18 months.

She had fallen from the stairs and had sprained her left knee 18 months previously. Since then, her symptoms had gradually worsened. Range of motion (ROM) in her left knee was 15° to 80° of flexion due to apprehension and pain around the medial patella. The lateral patellar apprehension test was positive.

**Fig. (1):**
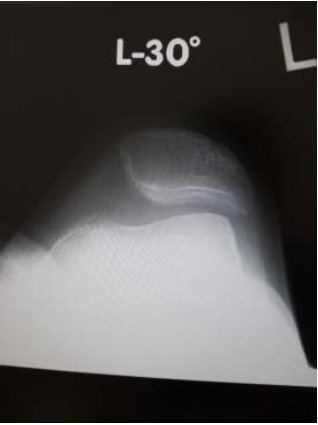
Roentgenogram showing lateral patellar subluxation.

**Fig. (2):**
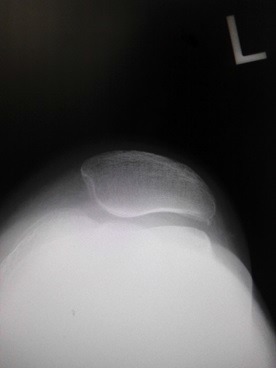
Roentgenogram taken prior to the injury shows no lateral patellar subluxation.

On initial examination, roentgenogram showed lateral patellar subluxation, a Q angle of 0°, congruence angle of −35°, and sulcus angle of 153° (Fig. **1**). By contrast, roentgenogram taken 2 years previously during a medical check-up of the knee had shown no remarkable findings. The Kellgren–Lawrence grade [6] for her knee had been 0, with a femorotibial angle of 175°, Q angle of 3°, congruence angle of 0°, and sulcus angle of 145° (Fig. **2**).

**Fig. (3):**
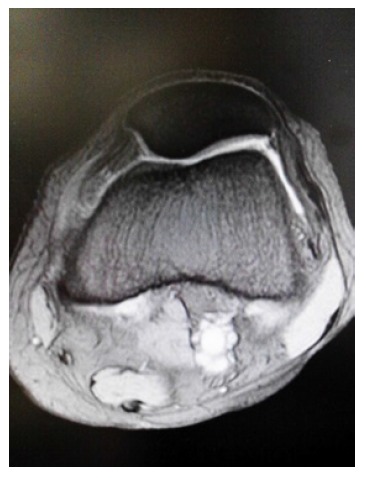
Magnetic resonance imaging showing a slack and torn medial patellofemoral ligament at the femoral attachment.

T2-weighted magnetic resonance imaging showed a slack and torn MPFL at the femoral attachment (Fig. **3**). Computed tomography (CT) showed 8° of tibial external rotation.

Her Kujala score [7] was 66 points, and Barthel index [8] score, which is used to qualify the activities of daily living (ADLs) among aged people, was 70 points (total score, 100 points).

**Fig. (4):**
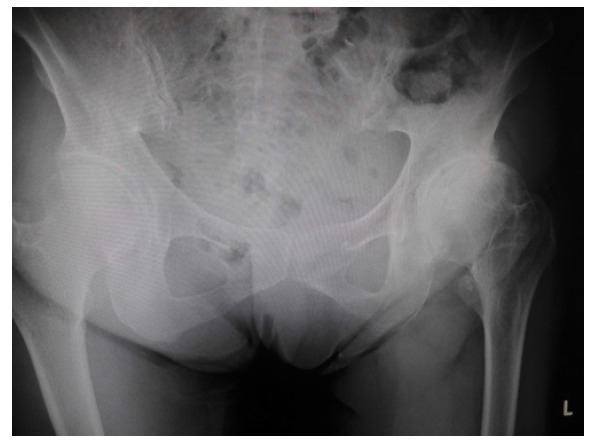
Roentgenogram of the hip joint shows a narrowing of the left joint space, which was diagnosed as left hip osteoarthritis.

After the initial examination, conservative therapy, including the application of a patella-medializing knee orthosis, and physiotherapy were started, but her symptoms of instability, pain, and giving way of the patella when standing up from a chair and climbing stairs did not resolve even after 3 months of follow-up. We noticed that her hip joint was externally rotated during walking, although she did not complain of any other symptoms except for those related to her left knee. However, roentgenogram of her hip joint showed narrowing of the left joint space, which was diagnosed as left hip OA (Fig. **4**). Hip ROM was 125°/75° of flexion, 40°/30° of abduction, and 45°/15° of internal rotation. The Japanese Orthopaedic Association (JOA) hip score [9] was 87 points (total score, 100 points), and the Harris hip score [10] was 88 points. The patient and her family confirmed that her gait had not changed for years.

Based on the aforementioned findings, we considered that she had asymptomatic left hip OA and had injured her left MPFL during the fall from the stairs; a valgus force had been applied to her left knee, with external rotation of her left hip joint.

We performed MPFL reconstruction and lateral release to reconstruct the primary stabilizer and to achieve proximal realignment of the patella.

## 3 OPERATIVE FINDINGS

The patient was placed under general anesthesia in the supine position. First, the surgeon made a conventional lateral parapatellar portal and a general intra-articular evaluation was performed. The patella was laterally subluxated in the extension position. The cartilage of the patella was slightly fibrillated, but subchondral bone was not observed. A degenerative horizontal tear in the posterior section of the medial meniscus was observed, and partial meniscectomy was performed. There were not any other lesions such as lateral meniscus tear or chondral lesion at the femorotibial joint. Then, lateral release from within the joint using scalpel was performed. After these procedures, the left leg of the patient was placed in the (Fig. **4**) position for semitendinosus (ST) and gracilis (G) tendon harvesting. A 3 cm oblique skin incision was made at the medial proximal tibial area, and the soft tissue was released. ST and G tendons were harvested using a tendon stripper (Smith & Nephew Endoscopy, Japan), and the tendon lengths were 240 and 220 mm. The diameter of the doubled ST and G tendon was 6 mm. A femoral bone tunnel was constructed at the MPFL anatomical footprint [4], which is located 10 mm dorsal and 5 mm proximal to the femoral medial collateral ligament footprint. A 2.4mm guide wire was inserted in the lateral, ventral, and slightly proximal direction to avoid vessel and nerve injury. A 4.5-mm ENDOBUTTON drill (Smith & Nephew Endoscopy, Japan) and 6-mm drill were used to create a bone tunnel with a 20-mm socket. The depth of the bone tunnel was 66 mm. We then passed the tendons through a 55-mm long ENDOBUTTON CL (Smith & Nephew Endoscopy Japan) to place them 11 mm within the bone tunnel. The free ends of the tendons were tethered using 2-0 TiCron (COVIDIEN, Japan). After preparing the graft, 3-cm longitudinal skin incisions were made at the patellar and femoral natural footprints of the MPFL, and the soft tissue was peeled off so that the graft could pass through the soft tissue tunnel smoothly.

**Fig. (5):**
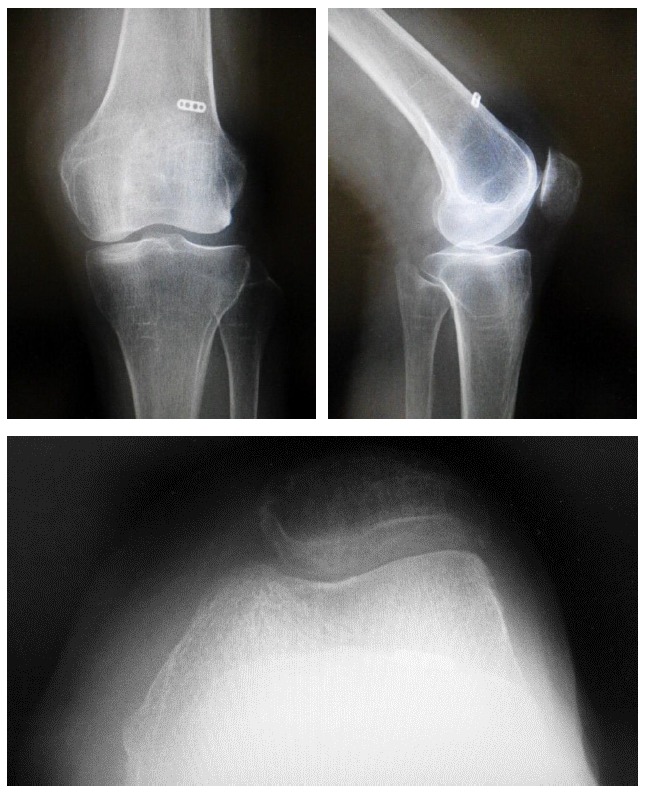
Postoperative roentgenogram taken after medial patellofemoral ligament reconstruction.

The graft was passed through the femoral bone tunnel using a 2.7-mm passing pin (Smith & Nephew Endoscopy, Japan). Flipping of the ENDOBUTTON device was confirmed on fluoroscopy. Subsequently, the graft was passed through the soft tissue tunnel to the patella, and the tethered free ends were sutured to the periosteum of the patella using two 1.9-mm Juggerknot anchors (Biomet Sports Medicine, Japan) at 60° of knee flexion, with the patella reduced to prevent lateral subluxation (Fig. **5**). The soft tissue was closed in layers, and a knee brace was applied to keep the knee in extension.

Knee ROM exercise was gradually started using a patella-stabilizing orthosis on the first postoperative day. In addition to knee rehabilitation, hip ROM rehabilitation and muscle strength training were also started to prevent the patient from walking with her left hip externally rotated. Weight-bearing walking was permitted at 1 month after surgery. Knee ROM improved to 0° of extension and 120° of flexion. Left hip ROM also improved from 70° to 90° of flexion and from −5° to 0° of extension, but internal rotation remained at 15°, as observed preoperatively. However, she could walk without external rotation of her left hip. Her Kujala score improved from 66 to 97 points, and the Barthel index score improved from 70 to 100 points. CT showed that the bone tunnel was placed at the anatomical footprint of the MPFL. She did not develop PFJ OA or had any recurrence of symptoms at 5 years postoperatively.

## 4 DISCUSSION

Case reports of patellar instability and patellar dislocation among young women with joint laxity are common [2, 3]. However, among aged populations, cases of total knee arthroplasty (TKA) for knee OA with permanent patellar dislocation [11] or lateral patellar dislocation after TKA are often reported [12, 13]. Accordingly, the authors concluded that the causes of lateral dislocation of the patella after TKA are component malpositions of an anterior, internally rotated, and medial femur; internally rotated tibia; and lateral patella. Only a few cases of MPFL reconstruction for patellar dislocation after TKA in the elderly have been reported [14]. The authors then ruled out component malrotation and limb alignment disorders using CT and concluded that the cause was not component malposition but medial support failure due to a medial parapatellar approach. In this case, the patient did not show knee OA on roentgenogram, and the joint surface findings on arthroscopic evaluation were Koshino grade 1 [15] and International Cartilage Repair Society classification grade 2 [16].Therefore, we performed proximal realignment and arthroscopic lateral release instead of TKA. We used a doubled ST and G tendon autograft with a diameter of 6 mm, which is almost the same as that in young women. We were concerned regarding the failure strength of the flexor tendons because the patient was an old female. Nevertheless, no recurrence occurred. The patient had left hip OA without hip pain. Some reports have described that in patients with advanced hip OA, external rotation of the hip joint tends to occur, and the pelvis are easy to take stoop [17]. We supposed that the patient had injured her MPFL when she fell down the stairs and landed on her left foot, during which a valgus force was applied to her left knee because her left hip leaned to compensate for the gluteus medius dysfunction, similar to that observed in Duchenne muscular dystrophy. Therefore, we considered that there was a risk of recurrence if the gluteus medius dysfunction and walking posture with external rotation of her left hip were not treated. We planned physical therapy without performing total hip arthroplasty (THA) because the patient did not complain of left hip pain and did not exhibit ADL deterioration. However, patients with traumatic MPFL injuries complain of symptomatic hip OA, MPFL reconstruction with THA should be considered.

## CONCLUSION

In an elderly female, we performed MPFL reconstruction using an ST and G tendon autograft for patellar instability due to trauma from a fall. We supposed that hip dysfunction caused by hip OA had played a role in the injury; therefore, we performed not only MPFL reconstruction but also physical therapy for the hip joint, through which the patient made satisfactory progress without developing PFJ OA or recurrence of symptoms during 5 years of follow-up.
